# Feasibility of Indonesia Family Life Survey Wave 5 (IFLS5) Data for Air Pollution Exposure–Response Study in Indonesia

**DOI:** 10.3390/ijerph17249508

**Published:** 2020-12-18

**Authors:** Dwi Agustian, Cut Novianti Rachmi, Noormarina Indraswari, Anna Molter, Melanie Carder, Fedri Ruluwedrata Rinawan, Martie van Tongeren, Driejana Driejana

**Affiliations:** 1 Department of Public Health, Faculty of Medicine, Universitas Padjadjaran, Jalan Eyckman No. 38, Bandung 40151, Indonesia; cutnovianti@gmail.com (C.N.R.); noormarina@unpad.ac.id (N.I.); f.rinawan@unpad.ac.id (F.R.R.); 2 Centre for Occupational and Environmental Health, Division of Population Health, Health Services Research and Primary Care, School of Health Sciences, Faculty of Biology, Medicine and Health, The University of Manchester, Oxford Road, Manchester M13 9PL, UK; anna.molter@ucd.ie (A.M.); Melanie.Carder@manchester.ac.uk (M.C.); 3 Department of Geography, School of Environment, Education and Development, Faculty of Humanities, The University of Manchester, Oxford Road, Manchester M13 9PL, UK; 4 Spatial Dynamics Lab, School of Architecture, Planning and Environmental Policy, University College Dublin, Richview, D14 E099 Dublin, Ireland; 5 Centre for Epidemiology, Division of Population Health, Health Services Research and Primary Care, School of Health Sciences, Faculty of Biology, Medicine and Health, The University of Manchester, Oxford Road, Manchester M13 9PL, UK; Martie.J.Van-Tongeren@manchester.ac.uk; 6 Air and Waste Management Research Group, Faculty of Civil and Environmental Engineering, Institut Teknologi Bandung. Jalan Ganesha No. 10, Bandung 40132, Indonesia

**Keywords:** self-reported, urban air pollution, spatial analysis, air pollution health impact, Jakarta

## Abstract

Background: Air pollution is an important risk factor for the disease burden; however there is limited evidence in Indonesia on the effect of air pollution on health, due to lack of exposure and health outcome data. The objective of this study is to evaluate the potential use of the IFLS data for response part of urban-scale air pollution exposure–health response studies. Methods: Relevant variables were extracted based on IFLS5 documentation review. Analysis of the spatial distribution of respondent, data completeness, prevalence of relevant health outcomes, and consistency or agreement evaluation between similar variables were performed. Power for ideal sample size was estimated. Results: There were 58,304 respondents across 23 provinces, with the highest density in Jakarta (750/district). Among chronic conditions, hypertension had the highest prevalence (15–25%) with data completeness of 79–83%. Consistency among self-reported health outcome variables was 90–99%, while that with objective measurements was 42–70%. The estimated statistical power for studying air pollution effect on hypertension (prevalence = 17%) in Jakarta was approximately 0.6 (α = 0.1). Conclusions: IFLS5 data has potential use for epidemiological study of air pollution and health outcomes such as hypertension, to be coupled with high quality urban-scale air pollution exposure estimates, particularly in Jakarta.

## 1. Introduction

The United Nations has made a significant change in placing the major diseases and risk factors in its agenda during the Third High-level Meeting on Non-Communicable Diseases (NCDs) on 27 September 2018. Environment, particularly air pollution, is now the fifth risk factor, in addition to smoking, excessive consumption of alcoholic beverages, unhealthy eating patterns, and lack of exercise [1]. In fact, air pollution is increasingly recognized as an urbanization and industrialization challenge in many lower- and middle-income countries.

Indonesia is the fourth most populous country in the world. The population was nearly 262 million in 2017; projected to be 285 million in 2025 with over 50% and growing number of people living in urban areas [2]. Many of Indonesian urban areas are sprawling with associated environmental problems, including air pollution. The problem often relates to transportation, with the significant increase in number of private cars and motorcycles [3]. Currently, in total there are over 140 million motor vehicles in Indonesia, of which motorcycles steadily comprised about 80% of the total [4]. From 2001 to 2011, the transport sector portion of the total national fossil fuel consumption was almost doubled, from 45% to 80% [2]. This would be a significant emission source in addition to industry, hence the associated health impacts.

The impacts of air pollution exposure on short-term health effects such as asthma [5,6,7] and longer-term outcomes or chronic diseases such as cancer [8,9], hypertension and/or diabetes [10,11,12], and stroke [13,14] are well studied elsewhere. The national health statistics trends from 1990 to 2017 indeed showed an increase burden of degenerative or chronic diseases such as stroke, diabetes, hypertension, and other cardiovascular diseases [15]. Considering the similar trend in emissions, air pollution is likely to contribute to epidemiological and disease transition in Indonesia.

Despite the evidence of air pollution harmful effects on health in other countries, there have only been a few studies in Indonesia that directly linked air pollution exposure and health [16,17]. Some other studies in Indonesian context were done on air pollution levels [18,19], or health effects [20,21,22]. However, those studies were mainly focused on only one side of either exposure, or health outcomes.

Evidences from studies that link air pollution and health outcomes are important in order to develop and advocate the mitigation and control effort, based on the country specific findings. Such studies require high quality data on both air pollution exposure and health outcomes; that are collected by a proper design in a well-defined geographical or population area. The difficulty in investigating air pollution and health that arises from data lacking in Indonesia had been recognized by a previous study [23]. The government currently is expanding the air quality monitoring network, which will gradually close the gap in air pollution data. However, at the moment the availability of data on air pollution exposure is still limited. The extent of air quality monitoring network is relatively scarce and sparse, owing to the size of the country. In addition, considering most of the monitoring stations have now only been in operation for less than 10 years, it might be years to come before temporal/time series-based health impact analysis can be conducted.

While ample data for time-series analysis is not yet available, a more feasible solution to study the relationship between air pollution and health is through spatial analysis. With spatial analysis, data can be obtained in a relatively short time. The simplicity of spatial-based exposure estimates was successfully used in the EU ESCAPE Project (www.escapeproject.eu) in Europe. The EU ESCAPE project referred to the need of European-based estimates of air pollution health impacts in the EU, as the previous available estimates were based on North American exposure–response relationships. To provide such estimates, the project performed studies on refined exposure assessment, using the spatial modelling technique of Land Use Regression in 22 countries within Europe [24,25].

The EU ESCAPE methodology could be a promising solution for the development of air pollution exposure–response estimates and compliance in Indonesian cities. The EU ESCAPE exposure modelling technique is adopted in the on-going project of UDARA (Urban hybriD models for AiR pollution exposure Assessment). UDARA is a multi-disciplinary, Indonesia-UK join research with the overall aim of developing a new approach for providing reliable exposure estimates.

Nevertheless, the lack of suitable data for undertaking epidemiological studies of air pollution does not only occur to exposure, but also to health outcomes. For that reason, the Indonesian Family Life Survey (IFLS) by RAND Corporation (rand.org) is considered as a potential source of health outcomes data to be matched with the spatially-resolved exposure data such as derived from the land use regression (LUR) model. IFLS is a longitudinal cohort dataset, which is a representative of approximately 83% of the Indonesian population [26]. The first survey (Wave 1) was undertaken in 1993, followed by Wave 2, 3, 4, and 5 in 1997, 2000, 2007 and 2014, respectively.

IFLS data have been used for a number of health-related studies, e.g., to investigate the multi-morbidity patterns and their prevalence [27]. Sohn [28] used the IFLS data to investigate effects of education on smoking habits in youth, while Christiani et al. [29] studied whether Indonesian women living in major cities have a higher risk of chronic conditions.

Some studies specifically used IFLS to investigate the impacts of air pollution on diseases. For example, Silwal and McKay [30] showed that individuals living in households that used firewood as fuel for cooking have lower lung capacity. Most often, studies had taken the time of significant air pollution episodes in Indonesia due to biomass burning (BB). Forest fires (FF) in 1997, which coincided with IFLS Wave 2, was used in a number of studies as the baseline to investigate FF-associated health impacts [31]. Other studies used IFLS Wave 3 database from surveillance done in 2000, or contrasting it with Wave 1 in 1993 [31,32,33,34,35]. For exposure estimate, these studies mostly exploited information from global sources, e.g., NASA satellite images as the proxy of air pollution levels. Frankenberg et al. [31] found statistical evidence that linked 1997 FF to increased difficulty of daily living activities and negative impacts on respiratory and general health. Kim et al. [32,33,34] utilized TOMS’s aerosol index in a wide area of Indonesia affected by biomass burning (BB) in 1997. Their studies coupled global air quality proxies with IFLS data of the 2000 and 2007 waves. Kim et al. [33] found that the episodic shock of air pollution were significantly linked to clinical depression in women in the BB area, and shortened working hours of workers up to ten years after the air pollution shocks [34]. Recent study by Rosales-Ruedo and Triyana [35] based on the five Waves of IFLS found the impacts of early-life exposure during 1997 FF to exposed children were long-lasting towards their adulthood.

These studies had suggested relationships between increasing air pollution to various physiological and mental health responses. All provided statistical evidences of air pollution negative impacts to chronic health and welfare in Indonesia; however, all were on a countrywide-scale of the national population. Kim et al. [32], raised two important points on exposure and health outcomes used in the studies. Firstly, air pollution data was generated from global remote sensing images that covered the large area of Indonesia, therefore it was lack of small scale spatial concentration; hence, also, variation in related impacts. Secondly, the implication of air pollution being studied including the economic loss only considered the short term of episodic event, therefore overlooked the negative impacts due to low but prolonged exposure typically observed in populated urban areas.

In order to quantify its disease burdens and develop mitigation strategies, there is a pressing need for exposure and health outcomes data in a local scale. The exposure estimates then can be used to study the health effects of air pollution in urban populations in Indonesia. To investigate the relationship, the small-scale spatial variation that exists in air pollution level requires health outcomes on a similarly fine scale. The fine-scale resolution exposure–response study is important for developing air quality management strategies. Likewise, planning and implementation of air quality management policy. This is even more important in Indonesia with the existence of the Local Autonomy Bill, where the authority to develop such policy is placed on local/city governments. However, unlike in Europe where health data for such purposes could be derived from on-going cohort studies, in Indonesia the availability of health outcome data fit for the aforementioned purpose is also challenging. To fill in this gap, we explored the possibility of using IFLS data for health response part for such study.

The coverage of IFLS data might allow geographical clustering of health-related information at different geographical resolutions, and therefore could potentially provide health outcome (and confounder) data for a spatial-based air pollution epidemiological study. This paper reviewed the health outcome data collected by IFLS in terms of its completeness, geographical distribution, and how IFLS can be used for local-scale spatial epidemiological studies. The health outcome data were examined by a comparison of self-reported and objective measurements; and potential confounder data (for example smoking and other lifestyle factors). The extracted IFLS data furthermore are expected to be coupled with the on-going LUR modeling in The UDARA project. Moreover, the results of this study may be utilized for future recommendation in setting the adequate infrastructure to monitor health impacts of air pollution and the development of air quality management strategies in Indonesian cities.

## 2. Materials and Methods

Within UDARA there are specific work-packages (WPs) dedicated to air quality modelling and monitoring. Similar to EU ESCAPE, in LUR modelling, the air quality data as one of the model input is based on measurements using low-cost instruments. Low-cost methods were commonly used in spatial-based monitoring and LUR modelling [24,25,36,37,38]. In UDARA, gaseous NO_2_ and O_3_ were measured with passive samplers [39,40] and PM_10_, PM_2.5_, PM_1.0_ were measured with low-cost sensor (LCS) based on light scattering, similar to the study by Pope et al. [41].

Application of low-cost methods allowed measurements to be done in many sampling locations, providing ample data for air pollution spatial gradient and spatially-resolved air pollution modelling, such as land use regression. Land use regression models and its hybrid version with remote sensing and chemical transport models, will then be developed and used to obtain air pollution measures, with spatial and temporal resolution. The air pollution study will produce annual average concentrations, which will be fit for exposure assessment of chronic diseases, e.g., health outcome data obtained from IFLS.

IFLS, originally conducted in 1993, is an ongoing longitudinal survey targeting the original respondents (and their descendants) in each of the subsequent surveys (1997, 2000, 2007, and 2014). All of the data from the 5 waves have been publicly released. Each wave has a number of books for different type of data. The IFLS5 collected data from 58,304 participants comprised of 32,507 adults, 13,895 adolescents, and 11,902 children. Data were collected using a strict protocol and include extensive individual, household, and community level measurements on a wide-range of demographic, socio-economic, lifestyle, health, and other factors. Detailed information about IFLS is available elsewhere [26].

**Figure 1:**
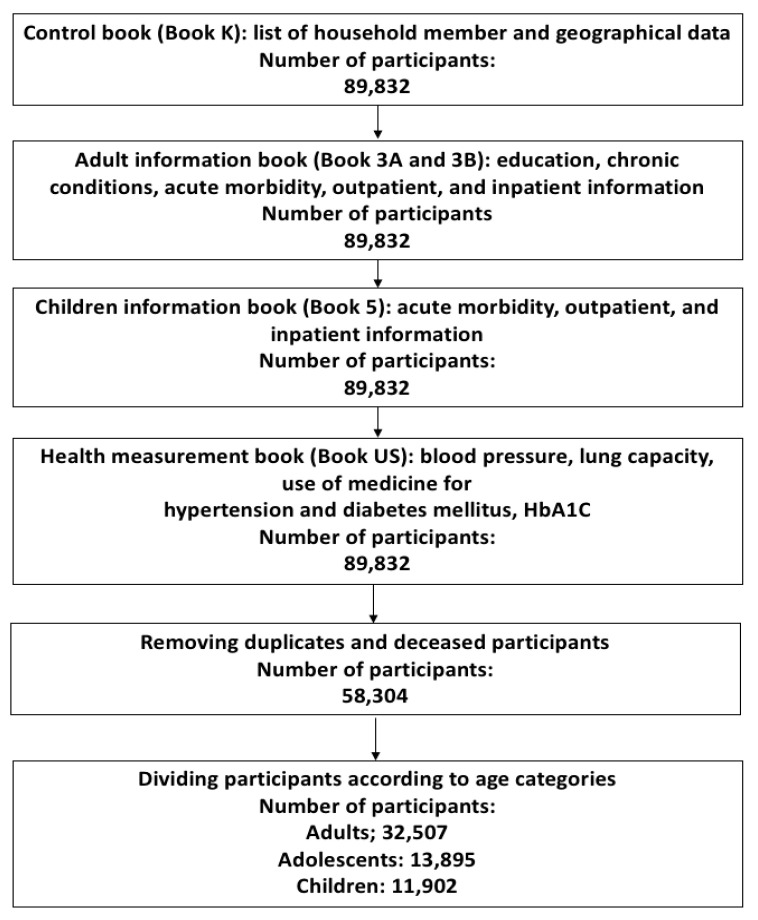
Data source, extraction, and merging steps.

Four provinces, namely Jakarta, Sumatera Utara, Sumatera Selatan, and Kalimantan Selatan, were selected based on the historical exposure of biomass burning, higher number of IFLS respondents [26], and air pollution data availability potential. In this study we use data from of the last wave (IFLS5) that were taken in 2014. The data required for our study were extracted from control book (Book K), adult information book (Book 3A and 3B), children information book (Book 5) and health measurement book (Book US), with the merging steps illustrated in Figure 1.

After merging all the books, removing duplicates and deceased participants, we divided participants into three age categories, namely adults (25.0 years and older), adolescents (10.0–24.9 years), and children (0–9.9 years). This age grouping was based on the latest age definition of adolescent by Sawyer et al. [42]. Variables used for adults were derived from Book K, Book 3A, Book 3B, and Book US. The dataset for children were derived from Book K and Book 5. Since IFLS originally only categorized the adult participants as 15 years and older, while children as less than 15-year old, we matched variables from Book 3A, Book 3B, and Book 5 as well as other variables from Book K to determine adolescent category.

An initial screening exercise was undertaken whereby the individual questions asked in each of the seven books were reviewed and assessed using the following criteria: (1) does the answer to the question provide a health outcome measure and (2) has this health outcome measure been consistently linked with air pollution in studies elsewhere. For those questions with a positive response to (1) and (2) further analysis was carried out in relation to the data completeness, geographical distribution of the outcome measures, and the frequency of outcome.

The individual questions in each of the 7 books of IFLS5 were reviewed and assessed by asking (1) has the information provided by this question been shown to be a potential confounder in studies of air pollution and health? For those questions with a positive response further analysis was carried out in relation to data completeness.

We included data on self-reported measures on hypertension, diabetes mellitus, asthma, and objective measures (Blood Pressure, HbA1c, Peak Expiratory Flow/PEF). Book 5, where the self-reporting variables for children’s symptom came from, was administered to all household members younger than age 15. Children 11–14 were allowed to answer for themselves; an adult (usually the mother) answered for children younger than age 11. The blood pressure was performed for age ≥15 years three times (left arm, right arm, and left arm) following IFLS5 standard protocol. The mean of the blood pressure from the three measures was used in this paper. Lung capacity was measured as Peak Expiratory Flow (PEF) in L/min units (liters per minute) for members 9 years and older by a Personal Best Vitalograph Peak Flow Meter. Finger pricked were conducted for blood sampling and used for HbA1c level examination in adult population to measure the risk of diabetes. To assess the consistency of the self-reported measures, we calculated agreement between self-reported measures and objective measures and agreement between two self-reported related questions.

All analyses were performed using STATA Data Analysis and Statistical Software version 13 and R version 3.6.0. We conducted frequency tabulations to describe distributions. Results are presented as numbers and prevalence in percent along with confidence intervals. The data extraction procedure flow diagram is presented in Figure 1.

We analyzed the statistical power of a potential air pollution exposure–response study using IFLS data. For this purpose we assumed that a cross-sectional study design would be used. We used hypertension in adults as an example of a health effect of interest; therefore, the power calculation was based on a logistic regression model. The prevalence of hypertension in IFLS5 was used as the prevalence at the mean exposure and the prevalence at one standard deviation above the mean exposure was calculated based on findings from a previous study in China [10].

## 3. Results

Table 1 shows general information on subject characteristics as well as the respective total numbers and their percentages. We gathered data of 58,304 subjects from IFLS Wave 5, with balanced male:female ratio, representing the national distribution. More than half of the subjects were adults (55.8%) and the rest of the subjects were either children (20.4%) or adolescent (23.8%). Among the provinces that were selected from this study, Sumatera Utara held the highest number of the sample population (4953) compared to that of Jakarta, Sumatera Selatan, and Kalimantan Selatan.

**Figure 2:**
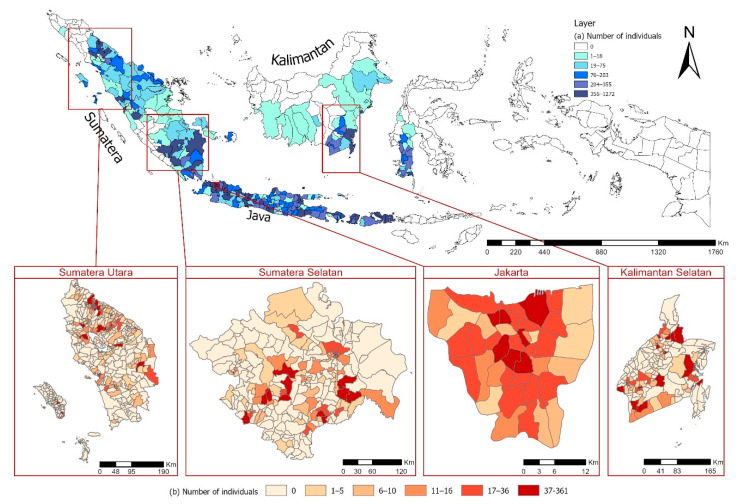
IFLS Wave 5 (**a**) Total number of individual surveyed per district; (**b**) subject density per sub-district at each province.

Although the highest number of respondent was found in Sumatera Utara, it was distributed into 29 districts (cities/regencies), while in Jakarta the survey only covered 5 districts (cities) out of 6 districts (cities/regencies). Therefore, in terms of average number of subjects per district, Jakarta was the highest. This implicates a denser spatial distribution of subjects compared to that in other selected provinces. The geographical distribution of the respondents and the health measures are shown in Figure 2, which supports the results of Table 1. The data is presented based on characteristics of sex, age group, and province, with district within province as the geographical unit (Figure 2a), and number of individuals within sub-districts (Figure 2b).

Figure 2a show that the highest number of survey subjects were in Java, Sumatera, and Kalimantan. As we look for spatial distribution of subjects in the selected provinces, in Figure 2b the number of subjects were mapped based on sub-district level. Figure 2b clearly shows that only Jakarta seems to have large number and evenly distributed respondents over the five districts. In other provinces which have more districts, most of sub-districts only have small number of survey subjects and some sub-districts have none (ranges of number of subject per district are provided in Supplementary Table S1).

### 3.1. Review of Data Completeness and Prevalence Data

Prevalence was taken both as self-reported data and objective measurement data. The identified potential health outcomes, self-reported data completeness and number of respondents with the outcome in question are shown in Table 2. At all population, variables were defined as “Breathing Difficulty” (consisted of “Wheezing” and “Fast Breathing”), “Cough”, “Hospitalization”, and “Outpatient Visits”. In addition to that variables, for adult population there were also data on “Hypertension”, “Stroke”, “Heart Problem”, and “Asthma”. The completeness of health outcome variables related with air pollution varied between 79.2% (outpatient visit) to 100% (wheezing and fast breathing among subjects with breathing difficulty). The completeness of less than 100% means that the respective data for the subject were not available (completeness referred to the answer of “Yes”, “No” and “Do not know” in all age group and all health outcome variables). Although the densities of the survey subjects per district in Jakarta were the largest, Jakarta data consistently showed the lowest response rates, on average 3–6% lower than that of other provinces.

In terms of prevalence of the health symptoms variables, “Cough” consistently had the highest prevalence (39.4–51.3%) in all population as is shown in Table 3. Furthermore, among chronic conditions in adult population surveyed by IFLS, “Hypertension” had the highest prevalence and number of cases. Although all response completeness of “Breathing Difficulty” were less than 100%, once the respondents complete the answer, at all age group, the cause of “Breathing Difficulty” either “Wheezing” or “Fast Breathing” were answered. For the chronic conditions in adult age, the respondents who did not give information, were found systematically missed all questions for unknown reasons, as were shown by the same number of subjects (and percentages) for all self-reported chronic condition in adult age. Although they were not exactly the same, similar percentages were also found in “Cough”, “Hospitalization”, and “Outpatient Visits”.

At provincial level, the number of subjects seems reasonable, still allowing detection of health outcome with low prevalence (see Table 2 and Table 3). This can be seen from previous studies that indeed had successfully demonstrated the link of IFLS health outcomes with aerosol index taken from satellite images as the proxy of FF smog, that usually have meso-to-macro-scale space dimension [31,32,33,34,35]. However, for exposure–response study, small-scale exposure concentration variation is likely to exist, particularly for the case of non-episodic urban pollution.

Not all self-reported data were accompanied with objective measurement data. Only 79% of the adult subjects had complete data in terms of both self-reporting data and objective measurement. The percentage was the lowest in Jakarta (74%), while three other provinces were ≥80% (see Table 3). The original data of objective measurement in IFLS data were in the forms of diastolic and systolic blood pressure levels for “hypertension”; HbA1C levels for Diabetes Mellitus (DM); and Peak Expiratory Flow (PEF) levels for respiratory function. We applied cut off levels for two objective measurements to determine its agreement to self-reported answers; which were 140 and 90 mmHg for systolic and diastolic hypertension, respectively; and HbA1C level of >6.5 for DM. Low PEF level may be correlated with Chronic Obstructive Pulmonary Disease (COPD) or Asthma status at the time of examination.

### 3.2. Comparison of Self-Reported and Health Measurement Data

IFLS gathered data from self-reported (through interview), physical, and laboratory examination. In Wave 5 most of the data were self-reported, as it is the most practical and cost-efficient method in a large-scale survey such as IFLS. It is essential that the inference of health outcome prevalence be supported by certainty that health outcome data derived from self-reported is a valid one. The value of self-reported data is important to be evaluated, because of its subjective nature and could increase measurement bias. We investigated the potential bias of self-reported outcomes by comparing those outcomes with available objective measures. Agreement was calculated by comparing two different questions on self-reported data that represent the same health outcomes (Table 4). Agreement is defined by consistent answers between the two Questions. For example, if a subject had hypertension, he should answer “Yes” for both Question 1, “Are you now taking the following treatment to treat hypertension and its complication?” and Question 2, “Have a doctor/paramedic/nurse/midwife ever told you that you had hypertension?” Table 4 shows that consistency for “Diabetes Mellitus” was the highest (on average of 98.57%) and the lowest was for “Asthma” (on average of only 70.32%).

In Table 5, for the comparison of self-reported questions with the objective measurements, we found that the agreement was around 42% for “Systolic Blood Pressure” and about 70% for “Diabetes Mellitus”.

Table 6 shows the estimated sample sizes that would be required to achieve statistical powers ranging from 0.6 to 0.9 in a potential study of air pollution exposure and hypertension in IFLS. For example, the results show that 8703 study participants would be required to detect an effect of PM_2.5_ exposure on hypertension with a power of 0.8 at a 5% significance level. The table shows ample sizes required to detect the effect of air pollution exposure on hypertension with a range of statistical powers (1–ß) and two alpha levels. Estimates are based on an odds ratio 1.07 for a 27.4µg/m^3^ increase in PM_2.5_ [10].

### 3.3. Review of Potential Confounder Data

For all health outcomes related to air pollution that we identified in IFLS5 data, we reviewed and collected potential confounders. The confounder variables in IFLS are listed in Table 7.

Table 7 lists variables identified within the IFLS that have been shown to be confounders and/or effect modifiers in epidemiological studies of the impact of outdoor air pollution on health. Although the specific requirements will depend on the type of study being undertaken, it is apparent that the IFLS contains individual and/or household level information for a number of key potential confounders/effect modifiers. Additionally, overall, the level of data completeness for the identified variables was very high, typically > 90%.

#### 3.3.1. Demographics

Individual level factors such as age, sex, height, weight, ethnicity, and marital status have been shown to be potential confounders/effect modifiers in epidemiological air pollution/health studies [23,43]. Over 99% of the adults participating in IFLS Wave 5 provided information on age, sex, and marital status with slightly lower proportions providing information on height and weight (90%) and ethnicity (87%).

#### 3.3.2. Smoking Status

Exposure to tobacco smoke (directly and/or indirectly) is an established potential confounder/effect modifier in air pollution epidemiology [44]. The IFLS contains a number of individual level questions relating to smoking status of the adults surveyed including questions related to both current and past smoking status, type of tobacco products used, daily/weekly consumption levels, and the age of starting and (if applicable) stopping smoking (data completeness >90% for these specific questions). These data can also be combined with data recorded elsewhere, for example the current age of the individual, to enable measures such as life time tobacco exposure (pack years) to be calculated or to estimate (indirect) exposure for children living within the same household. The IFLS also contains a household level measure of tobacco consumption (total household expenditure on tobacco in previous week) the responses from which could be used to cross-validate the individual level responses. Ninety nine point two percent (99.2%) of adults resided in households for which this information was provided.

#### 3.3.3. Diet

Although diet may not directly related with respiratory outcomes, it may also potentially confound/modify the effect of air pollution on certain health outcomes, such as hypertension [45]. The IFLS contains individual level questions about the types (by categories) and frequency of foods eaten the previous week to which over 99% of the adults surveyed in IFLS5 provided information. The IFLS also contains household level questions about consumption (total household expenditure on each food type in previous week) which as for smoking, could be used to cross-validate the individual level responses. Seventy five point four percent (75.4%) of adults resided in households for which this information was provided. There is also a household level question about alcohol consumption (total household expenditure in previous week) with 96.4% of adults residing in households for which this information was provided (there are no individual level questions about alcohol consumption in the IFLS).

#### 3.3.4. Socio-Economic Status and Other Exposure

Other sources of exposure to air pollutants such as the home or work environment may also need to be considered. The IFLS collects household level data on a key source of pollutants in the home environment, i.e., the type of stove used for cooking with 99% of the adults surveyed residing in a household for which this information was provided. The IFLS also includes a number of questions about current and past (nine years) employment history with 99% of (employed) adult respondents providing information about their type of employment. These data combined with other IFLS questions about the type of activities carried out and duration of employment/hours worked could facilitate occupational exposure estimates for different pollutants.

## 4. Discussion

From this study we found that IFLS5 data contained variables on cardiovascular (hypertension, stroke, and heart problem) and respiratory (breathing difficulty, wheezing, and cough) conditions. These variables are considered as potential health outcomes specifically related to air pollution that can be utilized for epidemiological studies. In addition, there are some more general health measures such as recent hospitalization and outpatient visits that were less useful since there was no specific information regarding the diagnosis information or the chief complains underlying the hospitalization or visit.

Based on self-reported variables, the prevalence of cardiovascular outcomes at province level were vary between 1.1% (stroke) and 17.7% (hypertension). Meanwhile, the prevalence of health outcome, based on physical examination, for example for hypertension condition, tended to be higher. For comparison, the Indonesian government conducted cross-sectional national survey called “Riset Kesehatan Dasar” (Riskesdas) [46]. Although the survey method were different, the nearest Riskesdas survey in 2013 [27] indicated similar results of a higher prevalence value for health outcome variable taken from physical examination. IFLS has its advantages compared to Riskesdas for chronic/long-term health effect as it is developed to provide longitudinal database built from cohort survey.

Air pollution epidemiological studies commonly require large populations to ensure the studies have sufficient power; hence, generally require routinely collected health data, or other large health databases. Examples of these data include national mortality records or hospital admission data. However, extensive changes to the Indonesian healthcare system since the mid-1990s (including the types of service available and the uptake of these services), and especially since decentralization in 2000 [47], would make the use of such data challenging. For the purpose of spatial exposure-response study at a fine scale such as land use regression (LUR), where the strength is on exposure spatial variation, the health outcome data should also be able to provide spatially distributed information. From the four provinces we explored in this study, we have examined data at finer scale such as district and sub-district. We found that Jakarta, with the largest number of IFLS5 respondents per district, could provide the most potential for such spatial analysis. While in the North Sumatera, South Sumatera and South Kalimantan the aggregated data showed sparser data, thus potentially induce variance instability due to the small number of samples per unit area of interest (district or sub district).

The large spatial variability in fact will considerably limit the usage of IFLS health outcome data for epidemiological study at district or sub district level. This confirms that the design of IFLS from the very beginning did not consider spatial resolution at finer level. Such level is often important in an epidemiological study to evaluate the health impact of air pollution for further used as the basis to develop air quality management strategy. The use of IFLS data had been revealed to be suitable for air pollution related studies at the provincial or national levels, as were done elsewhere [31,32,33,34,35,48]. Though these studies were successful in relating the health outcomes with some proxy of air pollutant levels, it may limit the impact of the evidence for policy advocacy at action level. The reason for that is that uncertainty may arise, such that it is difficult to interpret the conclusion to the districts/cities or even more at the sub-district levels. This is due to the fact that there might be significant local variations for both exposure level and health outcome prevalence.

Nevertheless, unlike the capital city of other provinces, Jakarta land area is a Province that consists of five cities (West, East, Central, North, and South Jakarta). The special administration as a Province is gained from its status as the national capital city. Its size and geographical context; however, still represents and often be treated as a city, e.g., in air quality management. Hence, the aggregate of IFLS of the five cities for the whole Jakarta area that covered 3752 subjects could be potential for such epidemiological study, by combining it with the small-scale spatial data of air pollution exposure such as provided in UDARA project.

The advanced methods to estimate air pollution level at a finer scale, such as land use regression and dispersion models provide more feasibility to reduce ecological bias in epidemiological study. However, this can be achieved if only health outcome data were available at similar resolution. From the fine resolution exposure assessment perspective leading to a specific study at district/city levels, this then could be translated into future investment for building an environmental public health and air pollution monitoring program. For instance, setting a various cohort population that is exposed to air pollution. For example, in school that is located in a heavy traffic area, a health monitoring program can be established to record any student absent due to related health problems cause by worsening air quality. Meanwhile, in the proximity of such setting, air pollution monitoring station could also be installed to measure and monitor the air exposure level.

IFLS data is unique and should be explored for maximum usage. The previous studies had demonstrated the usefulness of IFLS data in finding evidence and/or link of pollutant concentration shocks, mainly due to forest fire to a number of health symptoms and effects. These previous studies, however, only provide statistical evidences on a regional context of Indonesia as a whole. The use of satellite image as the proxy for pollutant concentration reduced their usefulness for further implementable policy development. The interests on inflated concentration during FF episodes and the use of satellite image for providing exposure data also drive the focus only to the impacts of particulates. IFLS, however, have potential to be used in exposure–response exercise in Jakarta, because the combined data of the cities in the whole Province will provide ample subjects for health outcomes data. The investigation on potential confounders’ data for traffic related air pollution [49] showed that exposure–response analysis could be correlated with spatial-air quality data obtained from ground measurements, not only for particulates but also for other pollutants such as oxides of nitrogen and ozone, to investigate the yearly average pollutant concentration on chronic diseases. The implication of this is that future health outcome measurement should be designed at this finer scale, at city or district level. Another strength of IFLS is the longitudinal data resulted from its cohort design. With the exposure spatial data derived from the LUR model or other high-resolution model, which could provide estimate of the respective time. Additionally, it also has potential for investigation of long-term effects of air pollution. However, this kind of study can only be conducted where the data density permits, such as in Jakarta.

## 5. Conclusions

Although it has limitation in statistical power, the IFLS has the most potential usage for high resolution/city scale epidemiological study in Jakarta. IFLS could be used for exposure response study in larger area/national level; however, with lower resolution. Currently a study is ongoing on modelling of exposures at a city scale in Jakarta to be coupled with the database extracted from this work. For future studies, we recommend for establishment of long-term air quality monitoring station and population cohort study in this area that can fulfil better statistical power and study design.

## Figures and Tables

**Table 1:** Subject characteristics of Indonesian Family Life Survey (IFLS) data Wave 5 (*n* (%)) and number of subjects per province and district.

Characteristics	Wave 5 *n* = 58,304 (%)	
Sex Female	29,719 (50.97)	
Age Group		
Children (<10 years)	11,902 (20.4)	
Adolescent (10–24.9 years)	13,895 (23.8)	
Adult (≥25 years)	32,507 (55.8)	
Province	Total	Average number of Subject Per district
Sumatera Utara (29 districts)	4593 (7.88)	158.4
Sumatera Selatan (16 districts)	2942 (5.05)	183.9
Jakarta (5 districts)	3752 (6.44)	750.4
Kalimantan Selatan (12 districts)	2538 (4.35)	211.5
Others	44,479 (76.29)	

**Table 2:** Completeness of Health Outcome data-number (%) of total individuals (by age group and selected provinces) for whom data are available.

Variables	Total IFLS5	Jakarta	Sumatera Utara	Sumatera Selatan	Kalimantan Selatan
**Children**	**N = 11,902**	**N = 747**	**N = 1103**	**N = 673**	**N = 527**
Breathing Difficulty	10,922 (91.8)	660 (88.4)	1018 (92.3)	617 (91.7)	475 (90.1)
* Wheezing	340 (100.0)	21 (100)	25 (100)	18 (100)	22 (100)
* Fast Breathing	340 (100.0)	21 (100)	25 (100)	18 (100)	22 (100)
Cough	10,922 (91.8)	660 (88.4)	1018 (92.3)	617 (91.7)	475 (90.1)
Hospitalization	10,921 (91.8)	660 (88.4)	1018 (92.3)	617 (91.7)	475 (90.1)
Outpatient Visit	10,622 (89.3)	660 (88.4)	1018 (92.3)	617 (91.7)	475 (90.1)
Peak Expiratory Flow	7934 (66.7)	222 (29.7)	7 (0.63)	16 (2.38)	527 (100.00)
**Adolescent**	**N = 13,895**	**N = 889**	**N = 1167**	**N = 710**	**N = 637**
Breathing Difficulty	12,061 (86.9)	753 (84.8)	1010 (86.6)	598 (84.3)	557 (87.5)
* Wheezing	643 (100.0)	56 (100.0)	52 (100.0)	37 (100.0)	25 (100.0)
* Fast Breathing	643 (100.0)	56 (100.0)	52 (100.0)	37 (100.0)	25 (100.0)
Cough	12,061 (86.9)	753 (84.8)	1010 (86.6)	598 (84.3)	557 (87.5)
Hospitalization	12,059 (86.8)	752 (84.6)	1011 (86.7)	598 (84.3)	567 (89.1)
Outpatient Visit	12,059 (86.8)	752 (84.6)	1011 (86.7)	598 (84.3)	557 (87.5)
Peak Expiratory Flow	9561 (68.8)	260 (29.2)	10 (0.9)	16 (2.3)	635 (99.7)
**Adult**	**N = 32,507**	**N = 2116**	**N = 2323**	**N = 1559**	**N = 1374**
Hypertension	26,998 (83.1)	1680 (79.4)	1983 (85.4)	1284 (82.4)	1172 (85.3)
Stroke	26,998 (83.1)	1680 (79.4)	1983 (85.4)	1284 (82.4)	1172 (85.3)
Heart Problem	26,998 (83.1)	1680 (79.4)	1983 (85.4)	1284 (82.4)	1172 (85.3)
Asthma	26,998 (83.1)	1680 (79.4)	1983 (85.4)	1284 (82.4)	1172 (85.3)
Breathing Difficulty	26,987 (83.1)	1680 (79.4)	1983 (85.4)	1283 (82.3)	1172 (85.3)
* Wheezing	2118 (100.0)	163 (100.0)	163 (100.0)	103 (100.0)	100 (100.0)
* Fast Breathing	2118 (100.0)	163 (100.0)	163 (100.0)	103 (100.0)	100 (100.0)
Cough	26,987 (83.0)	1680 (79.4)	1983 (85.4)	1283 (82.3)	1172 (85.3)
Hospitalization	26,972 (83.0)	1679 (79.3)	1983 (85.4)	1283 (82.3)	1171 (85.2)
Outpatient Visit	26,971 (83.0)	1677 (79.2)	1983 (85.4)	1282 (82.2)	1171 (85.2)
Blood Pressure	25,699 (79.1)	1559 (73.7)	1864 (80.2)	1244 (78.5)	1136 (82.7)
HbA1c level	5521 (17.0)	245 (11.6)	268 (11.5)	256 (16.4)	256 (18.6)
Peak Expiratory Flow	22,917 (70.5)	594 (28.1)	23 (1.0)	40 (2.6)	635 (46.2)

* The percentage calculated among those respondents who answered “Yes” to having breathing difficulty.

**Table 3:** Prevalence ^#^ of Health Outcomes from IFLS5 (N (%)).

Self-Reporting Variables	Total IFLS5	Jakarta	Sumatera Utara	Sumatera Selatan	Kalimantan Selatan
**Children**	**N = 11,902**	**N = 747**	**N = 1103**	**N = 673**	**N = 527**
Breathing Difficulty	340 (3.2)	21 (3.2)	25 (2.5)	18 (3.0)	22 (4.7)
* Wheezing	217 (63.9)	10 (47.7)	14 (56.0)	11 (61.2)	15 (68.2)
* Fast Breathing	249 (73.3)	15 (71.5)	19 (76.0)	13 (72.3)	17 (77.3)
Cough	5595 (51.3)	382 (57.9)	514 (50.5)	313 (50.8)	232 (48.9)
Hospitalization	525 (4.9)	31 (4.7)	37 (3.7)	25 (4.1)	13 (2.8)
Outpatient Visit	2557 (24.1)	178 (27.0)	241 (23.7)	129 (21.0)	69 (14.6)
**Adolescent**	**N = 13,895**	**N = 889**	**N = 1167**	**N = 710**	**N = 637**
Breathing Difficulty	643 (5.4)	56 (7.5)	52 (5.2)	37 (6.2)	25 (4.5)
* Wheezing	195 (30.4)	14 (25.0)	13 (25.0)	8 (21.7)	10 (40.0)
* Fast Breathing	583 (83.7)	42 (75.0)	40 (77.0)	31 (83.8)	24 (96.0)
Cough	4828 (40.1)	321 (42.7)	419 (41.5)	215 (36.0)	208 (37.4)
Hospitalization	455 (3.8)	33 (4.4)	28 (2.8)	21 (3.6)	15 (2.7)
Outpatient Visit	1579 (13.1)	91 (12.2)	133 (13.2)	75 (12.6)	40 (7.2)
**Adult**	**N = 32,507**	**N = 2116**	**N = 2323**	**N = 1559**	**N = 1374**
Hypertension	3989 (14.8)	298 (17.8)	283 (14.3)	194 (15.2)	231 (19.8)
Stroke	307 (1.2)	25 (1.5)	20 (1.1)	13 (1.1)	18 (1.6)
Heart Problem	523 (2.0)	50 (3.0)	46 (2.4)	16 (1.3)	14 (1.2)
Asthma	761 (2.9)	69 (4.2)	41 (2.1)	42 (3.3)	50 (4.3)
Breathing Difficulty	2118 (7.9)	163 (9.8)	163 (8.3)	103 (8.1)	100 (8.6)
* Wheezing	749 (35.4)	34 (20.9)	57 (35.0)	34 (33.1)	44 (44.0)
* Fast Breathing	1663 (78.6)	113 (69.4)	115 (70.6)	76 (73.8)	72 (72.0)
Cough	10,607 (39.4)	729 (43.4)	878 (44.3)	481 (37.5)	527 (45.0)
Hospitalization	1325 (5.0)	75 (4.5)	109 (5.5)	53 (4.2)	46 (4.0)
Outpatient Visit	5154 (19.2)	307 (18.4)	392 (19.8)	220 (17.2)	181 (15.5)
Measurement variables—Blood Pressure	N = 25,699	N = 1559	N = 1864	N = 1244	N = 1136
Hypertension Systolic	6453 (25.11)	402 (25.79)	414 (22.21)	291 (23.29)	350 (30.81)
Hypertension Diastolic	4615(17.96)	307 (19.69)	319 (17.11)	197 (15.84)	277 (24.38)
Measurement variable—Blood HbA1c level	N = 5521	N = 245	N = 268	N = 256	N = 256
Diabetes Mellitus	544 (9.85)	37 (15.10)	25 (9.33)	18 (7.03)	26 (11.16)

^#^ The prevalence was calculated with the denominator of valid responses only. * The percentage calculated among those respondents who answered “Yes” to having breathing difficulty.

**Table 4:** Consistency of health outcome data based on Agreement of Self-Reported Questions (%, 95% CIs).

Between Two Self-Reported Data			
Question 1	Question 2	Wave 5	
		Agreement (%)	*n* Total
**Hypertension**			
Are you now taking the following treatment to treat hypertension and its complication?	Have a doctor/paramedic/nurse/midwife ever told you that you had hypertension?	89.66 (89.32–89.99)	32,126
**Diabetes Mellitus (DM)**			
Are you taking medicine for diabetes?	Have a doctor/paramedic/nurse/midwife ever told you that you had diabetes or high blood sugar?	98.57 (98.44–98.7)	32,124
***Asthma***			
Did you ever experience wheezing in the last 4 weeks?	Have a doctor/paramedic/nurse/midwife ever told you that you had asthma?	70.32 (68.45–72.13)	2379
**Cholesterol**			
Are you taking medicine for cholesterol?	Have a doctor/paramedic/nurse/midwife ever told you that you had high cholesterol	96.22 (96.01–96.43)	32,122

**Table 5:** Agreement of Self-reported question and objective measurement in IFLS Adult Population.

Between Self-Reported and Objective Measurements			
Question	Objective Measurements	Wave 5	
		Agreement (%)	*n* Total
**Hypertension**			
Are you taking medicine for High Blood Pressure?	Systolic blood pressure ≥ 140 mmHg	42.37 (41.82–42.91)	31,675
Are you taking medicine for High Blood Pressure?	Diastolic blood pressure ≥ 90 mmHg	61.16 (60.62–61.69)	31,651
Have a doctor/paramedic/nurse/midwife ever told you that you had hypertension?	Systolic blood pressure	52.37 (51.79–52.95)	28,212
Have a doctor/paramedic/nurse/midwife ever told you that you had hypertension?	Diastolic blood pressure	64.55 (64.03–65.08)	31,646
**Diabetes Mellitus**			
Have a doctor/paramedic/nurse/midwife ever told you that you had diabetes	DBS;HbA1c > 6.5	69.98 (68.82–71.72)	6120

**Table 6:** Samples sizes required to detect the effect of air pollution exposure on hypertension.

	Alpha	
Power	0.05	0.1
(1–ß)	*n*	*n*
0.6	5075	3318
0.65	5804	3912
0.70	6626	4592
0.75	7574	5386
0.80	8703	6345
0.85	10,119	7562
0.90	12,052	9244

**Table 7:** Completeness of Confounder Data-number (%) of total individuals by age group for whom data are available.

Age Category	Variable	Level ^a^	Total IFLS
Children (0–9.9 years) N = 11,901	Height	I	10,497 (88.2%)
	Weight	I	10,579 (88.9%)
	Ethnicity	I	11,886 (99.9%)
	Ever attend school ^b^	I	5293 (91.2%)
	Highest level of education ^c^	I	3784 (99.9%)
	Type of food eaten ^d^	I	10,404 (87.4%)
	Frequency food type eaten ^d^	I	10,374 (87.2%)
	Income (previous year)	HH	11,552 (97.1%)
	Weekly expenditure on tobacco	HH	11,425 (96.0%)
	Weekly expenditure on alcohol	HH	11,483 (96.5%)
	Type of cooking stove	HH	11,525 (96.8%)
Adolescents (10–24.9 years) N = 13,895	Height	I	11,693 (84.2%)
	Weight	I	11,680 (84.1%)
	Ethnicity	I	13,883 (99.9%)
	Marital status	I	13,892 (99.9%)
	Ever attend school	I	12,097 (87.1%)
	Type of food eaten ^d^	I	11,722 (84.4%)
	Frequency food type eaten ^d^	I	11,719 (84.3%)
	Ever smoker ^e^	I	7256 (52.2%)
	Current smoker ^e^	I	7256 (52.2%)
	Age start smoking ^f^	I	1793 (98.6%)
	Tobacco consumption ^f,h^	I	1801 (99.0%)
	Duration of current employment ^i^	I	3598 (99.9%)
	Normal hours worked per week ^i^	I	3559 (98.8%)
	Total weeks worked per year ^i^	I	3568 (99.0%)
	Income (previous year)	HH	13,007 (93.6%)
	Weekly expenditure on tobacco	HH	13,196 (95.0%)
	Weekly expenditure on alcohol	HH	13,268 (95.5%)
	Type of cooking stove	HH	13,320 (95.9%)
Adults (≥25 years) N = 32,477	Height	I	25,661 (79.0%)
	Weight	I	25,644 (79.0%)
	Ethnicity	I	32,444 (99.9%)
	Ever attend school	I	27,132 (83.5%)
	Highest level of education ^c^	I	25,425 (99.9%)
	Type of food eaten ^d^	I	24,504 (75.5%)
	Frequency food type eaten ^d^	I	24,502 (75.4%)
	Ever smoker	I	27,009 (83.2%)
	Current smoker	I	27,009 (83.2%)
	Age start smoking ^f^	I	10,136 (94.1%)
	Age stop smoking ^g^	I	1507 (97.6%)
	Tobacco consumption ^f,h^	I	10,681 (99.2%)
	Primary occupation ^i^	I	20,869 (99.9%)
	Duration of current employment ^i^	I	20,782 (99.5%)
	Normal hours worked per week ^i^	I	20,675 (99.0%)
	Total weeks worked per year ^i^	I	20,690 (99.1%)
	Income (previous year)	HH	31,330 (96.5%)
	Weekly expenditure on tobacco	HH	30,551 (94.1%)
	Weekly expenditure on alcohol	HH	30,726 (94.6%)
	Type of cooking stove	HH	30,866 (95.0%)
	Total number of individuals		58,304

^a^ I = individual level data, HH = household level data; ^b^ as % of children 5 years and over (Number of individuals: total IFLS = 5773); ^c^ as % ever individuals who ever attended school (Adult: total IFLS = 25,427; Adolescent: total IFLS = 12,048; Children over 5: Total IFLS = 3786); ^d^ Individuals who provided information for at least one category of food types (maximum of 17 food type categories); ^e^ Individual level smoking data only available for individuals 15 years and over; ^f^ As percentage of ever smokers (Adult: total IFLS = 10,769; Adolescent: total IFLS = 1819); ^g^ As percentage of quit smokers (Adult: total IFLS = 1544; Adolescent: total IFLS = 131); ^h^ Daily consumption for self-rolled, cigarettes and cigars (excludes chewing tobacco and pipes) and/or weekly consumption for self-rolled, chewing tobacco and pipes (excludes cigarettes and cigars); ^i^ As percentage of currently employed (individual level occupation data only available for individuals 15 and over; Adult: total IFLS = 20,877; Adolescent: total IFLS = 3603).

## Supplementary Materials

The following are available online at https://www.mdpi.com/1660-4601/17/24/9508/s1, Table S1: Aggregated Data at District Level.
