# Supplementary material for: Feasibility of Indonesia Family Life Survey Wave 5 (IFLS5) Data for Air Pollution Exposure–Response Study in Indonesia
Source: Int J Environ Res Public Health. 2020 Dec 18;17(24):9508. doi: 10.3390/ijerph17249508 (PMC7766249; doi:10.3390/ijerph17249508)
Supplement: Supplementary file 1 [file ijerph-17-09508-s001.zip › Supplementary Tables.docx]

Supplementary Tables

Table S.1. Aggregated Data at District Level

|  | Total IFLS5 | Jakarta | Sumut | Sumsel | Kalsel |
| --- | --- | --- | --- | --- | --- |
| No of district | 295 | 5 | 29 | 15 | 12 |
| No of subject range per district | | | | | |
| Adult  Adolescent  Children | 1-791  1-291  1-244 | 262-487  96-203  78-185 | 1-358  1-221  1-179 | 4-303  2-142  2-126 | 7-261  5-128  1-119 |
| Health outcome variables, No of subject range | | | | | |
| Breathing Difficulty | 0-62 | 22-39 | 0-23 | 0-24 | 0-21 |
| *Wheezing | 0-21 | 5-9 | 0-11 | 0-11 | 0-11 |
| *Fast Breathing | 0-57 | 17-34 | 0-16 | 0-19 | 0-15 |
| Cough | 0-259 | 84-167 | 1-125 | 1-98 | 3-92 |
| Hospitalization | 0-65 | 20-38 | 0-41 | 0-19 | 0-13 |
| Outpatient Visit | 0-229 | 58-164 | 0-124 | 0-81 | 0-44 |
| Hypertension | 0-96 | 29-72 | 0-34 | 0-37 | 0-43 |
| Stroke | 0-10 | 1-9 | 0-4 | 0-3 | 0-4 |
| Heart Problem | 0-15 | 6-15 | 0-8 | 0-4 | 1-4 |
| Asthma | 0-19 | 9-19 | 0-10 | 0-13 | 0-12 |

*The number among those respondents who answered ‘Yes’ to having breathing difficulty
